# Direct Susceptibility Testing of Mycobacterium tuberculosis for Pyrazinamide by Use of the Bactec MGIT 960 System

**DOI:** 10.1128/JCM.03162-15

**Published:** 2016-04-25

**Authors:** Anne-Marie Demers, Amour Venter, Sven O. Friedrich, Gabriel Rojas-Ponce, Daniel Mapamba, Levan Jugheli, Mohammed Sasamalo, Deepak Almeida, Afton Dorasamy, Ute Jentsch, Mara Gibson, Daniel Everitt, Kathleen D. Eisenach, Andreas H. Diacon

**Affiliations:** a Molecular Biology and Human Genetics, Department of Biomedical Sciences, Faculty of Medicine and Health Sciences, Stellenbosch University, Cape Town, South Africa; b MRC Centre for Molecular and Cellular Biology, Department of Biomedical Sciences, Faculty of Medicine and Health Sciences, Stellenbosch University, Cape Town, South Africa; c Division of Physiology, Department of Biomedical Sciences, Faculty of Medicine and Health Sciences, Stellenbosch University, Cape Town, South Africa; d National Institute of Medical Research, Mbeya Medical Research Center, Mbeya, Tanzania; e Swiss Tropical and Public Health Institute, Basel, Switzerland; f Ifakara Health Institute, Bagamoyo, Tanzania; g KwaZulu-Natal Research Institute for Tuberculosis and HIV, Durban, South Africa; h Clinical Laboratory Services, Division of Wits Health Consortium and School of Pathology, Johannesburg, South Africa; i Global Alliance for TB Drug Development, New York, New York, USA; j Department of Pathology, University of Arkansas for Medical Sciences, Little Rock, Arkansas, USA

## Abstract

Pyrazinamide (PZA) is a key antituberculosis drug, yet no rapid susceptibility test is commercially available. PZA drug susceptibility testing (DST) was performed directly on sputum samples from 327 patients and compared with the indirect method by using the Bactec MGIT 960 system in the context of patient screening for participation in a drug trial. Compared to standard indirect PZA DST, direct DST was successful in only 59% of cases, but results obtained were highly accurate and available faster. Agreement between the direct and indirect methods varied from 90 to 100% in each laboratory. The median times for obtaining PZA results from the time when the specimen was collected ranged from 11 to 16 days for the direct test and 18 to 95 days for the indirect test across laboratories. The direct method is accurate and reproducible across laboratories. It can be expected to accelerate results in >50% of cases, but it cannot replace indirect DST for PZA. Phenotypic methods remain the gold standard for DST in drug trials. If future studies can optimize the method to decrease the number of uninterpretable results, direct MGIT DST could be the new phenotypic DST standard for clinical trials, providing more rapid detection of resistance to new drugs in experimental regimens.

## INTRODUCTION

Pyrazinamide (PZA) is a key antituberculosis (anti-TB) drug that has recently been shown to substantially enhance the activity of the novel agents bedaquiline (BDQ) and pretomanid (Pa) (PA-824) in murine models of TB (1–3) and phase II studies (4–6). Novel regimens based on the BDQ-PZA and Pa-PZA building blocks do not include isoniazid (INH) and rifampin (RIF) and are thus suitable for the treatment of multidrug-resistant TB (MDR-TB) (defined as TB resistant to at least INH and RIF).

PZA resistance in subjects with TB susceptible to INH and RIF is rare, i.e., 2% to 10% of non-MDR-TB cases in South Africa (7, 8) and elsewhere (9–11). In patients with MDR-TB, however, recent studies have found between 60% and 70% PZA resistance in South African trial centers (12). Clinical trials with a novel 3-drug regimen such as BDQ-Pa-PZA in MDR-TB patients would require confirmed PZA susceptibility because undetected PZA resistance exposes participants to the risk of acquisition of resistance to the other agents in the tested regimen.

Although rapid molecular susceptibility tests for detection of critical mutations directly in sputum samples are available for most first-line and the most important second-line agents, there is no commercial test for the rapid molecular detection of PZA resistance. The association of multiple mutations throughout the *pncA* gene with PZA resistance makes it difficult to design/develop a test for detection of PZA resistance (13). Phenotypic PZA testing in liquid culture medium is well established in clinical practice but lacks accuracy and reproducibility (14). Most reports cite problems of false PZA resistance detection with the MGIT 960 system, which is attributed to the inoculum concentration being too high (13). Another limitation of the phenotypic method is the long time to completion (15). This is due to the need to first grow a primary culture and then grow a secondary culture with PZA at the required concentration to determine phenotypic susceptibility. As an alternative to the indirect method, the test can be set up directly from the clinical specimen. This eliminates the initial culture, thus speeding up the availability of test results, but such an abbreviated procedure can lead to invalid results due to culture contamination or insufficient growth if the inoculum contains too few viable bacteria (15, 16). This method has been evaluated for INH and RIF but not yet for PZA.

We investigated whether PZA testing via the automated Bactec MGIT 960 liquid culture system (Becton Dickinson Diagnostic Systems, Sparks, MD) directly from sputum specimens is feasible and accurate and expedites the availability of PZA susceptibility results compared to the standard indirect method.

## MATERIALS AND METHODS

### Patient specimens and ethical approval.

Spot sputum specimens were collected from patients screened for eligibility to participate in a multicenter phase II trial of a novel anti-TB regimen containing PZA (6). Patients were adults from community clinics with newly diagnosed smear-positive pulmonary TB and no apparent concomitant illness or conditions that would make participation inadvisable. Prior to the study, one laboratory tested 31 consecutive specimens to validate direct MGIT drug susceptibility testing (DST) for PZA. For the study, five mycobacteriology laboratories performed screening tests on sputum samples, among which were acid-fast bacillus (AFB) smear microscopy, Genotype MTBDRplus version 2 and MTBDRsl (Hain Lifescience, Nehren, Germany), and direct MGIT DST for PZA (Becton Dickinson, Sparks, MD). These screening tests were performed in parallel as capacity allowed as long as the patient was still considered for participation based on microbiological or clinical criteria. Consequently, not all results were available for every subject. Although direct MGIT DST for PZA was to be performed on one specimen, two of the laboratories tested additional specimens (day −2 and day −1). Also, the intention was to test only smear-positive specimens; however, smear-negative specimens were tested, as the smear results were not always available before setup of direct DST. The institutional review boards of all the participating sites approved the study. Written informed consent for study participation was obtained from all patients.

### Bactec MGIT drug susceptibility testing methods.

Direct and indirect PZA susceptibility testing was performed as described previously by Siddiqi et al. and according to the manufacturer's instructions, respectively (15, 17). For the direct method, sputum specimens were processed by using the *N*-acetyl-l-cysteine–sodium hydroxide (NALC-NaOH) method, using a final concentration of 1 to 1.5% NaOH. The remaining pellet was resuspended in phosphate buffer (pH 6.8) up to a final volume of 2 ml and was used as the inoculum for PZA susceptibility testing. The resuspended pellet was diluted 1/10, and 0.5 ml was inoculated into the control tube (also containing polymyxin B, amphotericin B, nalidixic acid, trimethoprim, and azlocillin [PANTA] and the PZA enrichment supplement), while 0.5 ml of the undiluted resuspended pellet was inoculated into the tube containing 100 μg/ml PZA (and also containing PANTA and the PZA enrichment supplement). Tubes were incubated in the Bactec 960 MGIT instrument, according to the 21-day protocol for PZA susceptibility testing (17). Direct DST results from the MGIT instrument were recorded as susceptible (S), resistant (R), or uninterpretable (U). Indirect DST results were recorded as susceptible or resistant, since tests with uninterpretable results were repeated until valid results were obtained. If the direct or indirect PZA result was resistant, the PZA tube was checked visually for evidence of contamination, and a Ziehl-Neelsen stain and/or blood agar plate assay was performed to rule out contaminants. If contaminants were found, the result was reported as uninterpretable. Uninterpretable results were therefore classified as contaminated (including X400 errors reported by the MGIT instrument), growth failure (X200 errors due to insufficient growth, i.e., that the growth units of the control did not reach 400 within 21 days), or instrument failure.

### Data analysis and statistics.

The indirect result was regarded as the gold standard. Although there was a laboratory protocol, variations in the number and timing of direct and indirect tests performed were observed among laboratories. Laboratory 4 had duplicate indirect PZA results; only one result was considered for agreement analysis since duplicate indirect tests all gave the same results. For laboratories 2 and 5, direct tests were repeated up to 3 times on different screening specimens: only the pair where both direct and indirect tests were done on the same specimen was kept. For laboratory 4, direct tests were done on a separate specimen from that used in indirect tests; direct tests were done once, and indirect tests were repeated up to 2 times and paired as described above. No duplicate tests were done for direct tests or indirect tests in laboratory 3.

In order to calculate the direct MGIT success rate (reportable results), the reproducibility of replicate direct MGIT results, and the time to direct and indirect DST results, all test results were used. To calculate the agreement between the direct and indirect test results, the results were paired as described above. The time between the specimen collection date and the ultimate PZA result date was calculated regardless of whether the result was interpretable or not. No times were available for the validation study. All direct DST was performed within 48 to 72 h of specimen receipt in the laboratory, except for one laboratory. Sputum specimens were processed for MGIT culture within the same time frame. However, the time from determination of an M. tuberculosis-positive MGIT culture to the setup of indirect PZA DST varied.

Category agreement was calculated by dividing the number of categorical result matches (susceptible/resistant) by the total number tested (18). A chi-square test was used to compare proportions. Correlation was measured by using the Spearman rank correlation coefficient. SPSS software version 20 (SPSS Corporation, Chicago, IL) was used for all analyses.

## RESULTS

### Performance.

Validation was performed with 31 sputum specimens. Of these, 24 (77.4%) had reportable PZA results, 17 susceptible and 7 resistant, with an agreement of 100% between the results of the direct and indirect methods. The 7 uninterpretable results were due to growth failure in 6 cultures (85.7%) and contamination in 1 culture (14.3%) (Table 1).

**TABLE 1:** Summary of direct and indirect PZA test results^*a*^

Parameter	Value for laboratory					
	1 (validation study)	2	3	4	5	Total (not including validation study)
No. of patients	31	23	13	52	239	327
No. of indirect PZA tests	31	23	13	37	140	207
No. of direct PZA tests	31	47	13	51	287	398
No. of reportable direct PZA test results/total no. of direct tests done (%)	24/31 (77.4)	16/47 (34)	10/13 (76.9)	30/51 (58.8)	179/287 (62.4)	235/398 (59.0)
No. of uninterpretable direct PZA test results/total no. of direct tests done (%)	7/31 (22.6)	31/47 (66)	3/13 (23.1)	21/51 (41.2)	108/287 (37.6)	163/398 (41.0)
No. of uninterpretable direct PZA test results/total no. of uninterpretable results (%) caused by:						
X200 error (growth failure)	6/7 (85.7)	16/31 (51.6)	2/3 (66.7)	16/21 (76.2)	76/108 (70.4)	110/163 (67.5)
Contamination	1/7 (14.3)	15/31 (48.4)	1/3 (33.3)	5/21 (23.8)	31/108 (28.7)	52/163 (31.9)
Instrument failure					1/108 (0.9)	1/163 (0.6)
Direct results						
Median time (days) from date of collection to date of start of PZA testing (range)	NA	0 (0–0)	1 (0–3)	0 (0–3)	2 (0–35)	
Median time (days) from date of start of direct PZA testing to date of direct PZA test result (range)	NA	16 (3–29)	10 (7–21)	13 (5–25)	14 (1–25)	
Median time (days) from date of collection to date of PZA test result (range)	NA	16 (3–29)	11 (7–21)	13 (5–25)	16 (2–49)	
Indirect results						
Median time (days) from date of collection to date of start of PZA testing (range)	NA	88 (7–208)	29 (5–127)	48 (14–112)	11 (5–187)	
Median time (days) from date of start of indirect PZA testing to date of indirect PZA test result (range)^*b*^	NA	7 (5–13)	7 (7–14)	8 (6–16)	7 (5–19)	
Median time (days) from date of collection to date of PZA test result (range)	NA	95 (14–213)	40 (12–141)	59 (21–126)	18 (11–195)	
No. of pairs of direct/indirect results (interpretable results only)	24	10	10	10	109	139
No. of pairs in agreement; no. of pairs not in agreement	24 (17 S, 7 R); 0	10 (8 S, 2 R); 0	10 (10 S, 0 R); 0	9 (9 S, 0 R); 1 (direct R and indirect S)	105 (97 S, 8 R); 4 (1 direct R and indirect S; 3 direct S and indirect R)	134 (124 S, 10 R); 5 (2 direct R and indirect S; 3 direct S and indirect R)
% agreement	100	100	100	90.0	96.3	96.4

a Proportion of reportable direct PZA test results that are either smear negative, scanty, 1+, 2+, or 3+. S, susceptible; R, resistant; NA, not applicable.

b The difference between the PZA test start date and the PZA test result date does not include the time initially required to obtain a positive culture.

**FIG 1:**
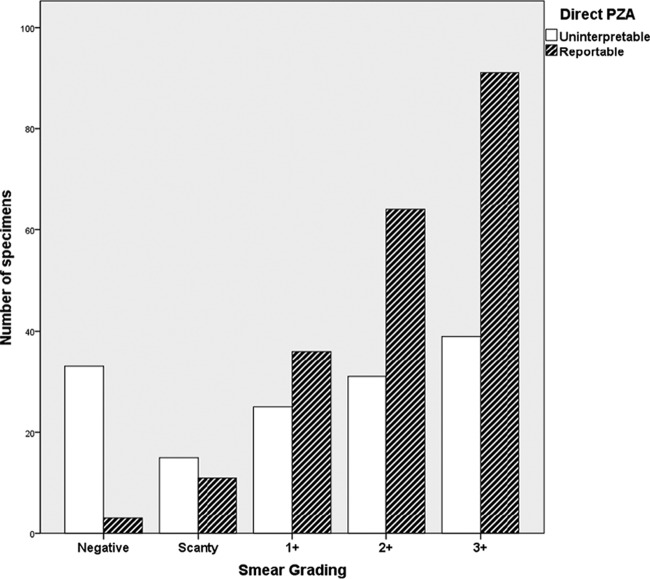
Reportable and uninterpretable PZA direct test results according to smear grading. The grading scale was based on WHO guidelines, as follows: negative (0 colonies/100 fields), scanty (1 to 9 colonies/100 fields), 1+ (10 to 99 colonies/100 fields), 2+ (1 to 10 AFB/field), or 3+ (>10 AFB/field).

PZA susceptibility testing was performed on sputum samples from 327 patients: 398 tests were performed by the direct method, and 207 were performed by the indirect method (Table 1). The direct PZA test results were uninterpretable for 163 samples (41.0%), varying from 23% to 66% among the five laboratories. Reasons for uninterpretable PZA direct testing results were growth failure for 67.5%, contamination for 31.9%, and instrument failure for 0.6% (for the distribution among laboratories, see Table 1). Of 398 direct PZA tests done, 348 had smear results available (87.4%): 36 were smear negative (10.3%) and were more likely to give an uninterpretable PZA direct testing result (33 uninterpretable results [91.7%]), compared to 312 positive smear specimens (110 uninterpretable results [35.3%]; chi square = 42.4; *P* < 0.001). This was due mainly to insufficient growth: 30 of the 33 uninterpretable results were due to X200 errors (91%). A correlation between smear grading and the proportion of uninterpretable PZA results was also observed (Spearman correlation = 0.298; *P* < 0.001) (Fig. 1).

### Agreement and reproducibility.

For all laboratories, an analysis of pairs (1 direct test result and 1 indirect test result per patient, as described in Materials and Methods) revealed that PZA resistance was detected in 12/139 (8.6%) pairs by the direct method and in 13/139 (9.4%) pairs by the indirect method. Of these 139 pairs, 134 were in agreement, and 5 were not, for 96.4% category agreement. Two of the discrepant results were resistant by the direct test and susceptible by the indirect test, while three were susceptible by the direct test and resistant by the indirect test (Table 1). No further testing was done to determine the true nature of this discordance.

Two laboratories performed direct tests in duplicate or triplicate. One had 20 sets of duplicate results (15 S/S and 5 R/R), showing 100% concordance. The other laboratory had 69% concordant results (9/13 [1 S/S/S, 1 R/R/R, and 7 uninterpretable {5 U/U and 2 U/U/U}]), 31% results with uninterpretable values (4/13 [1 S/S/U and 3 S/U/U]), and no discordant results. Only one laboratory performed indirect tests in duplicate: 11 results were concordant (1 R/R and 10 S/S), 1 result had and uninterpretable value (contaminated/susceptible), and there were no discordant results.

### Time to availability of results.

The median times to availability of results for each laboratory ranged from 11 to 16 days for the direct test, compared to 18 to 95 days for the indirect test (Table 1). Table 2 compares the numbers of direct PZA tests with results available (reportable or uninterpretable) 7, 14, 21, and 28 days after specimen collection. Variable times were observed, with >96% of the results being available at 21 days (i.e., the maximum duration for the MGIT PZA protocol) for 3 of the 4 laboratories. Such a comparison was not done for indirect PZA test results since the indirect tests were often not set up in real time.

**TABLE 2:** Numbers of direct PZA tests with results available (reportable or uninterpretable) 7, 14, 21, and 28 days after specimen collection

Laboratory	Day	No. of direct PZA tests with results available	No. of direct PZA test results available/total no. of direct PZA tests performed	% of direct PZA tests with results available
2	7	12	12/47	25.5
	14	11	23/47	48.9
	21	22	45/47	95.7
	28	1	46/47	97.9
3	7	2	2/13	15.4
	14	9	11/13	84.6
	21	2	13/13	100.0
	28	0	13/13	100.0
4	7	8	8/48	16.7
	14	20	28/48	58.3
	21	18	46/48	95.8
	28	2	48/48	100.0
5	7	7	7/269	2.6
	14	89	96/269	35.7
	21	100	196/269	72.9
	28	66	262/269	97.4

## DISCUSSION

In this multicenter clinical trial of a novel anti-TB treatment regimen, we compared PZA resistance testing performed directly on sputum specimens from untreated patients with indirect testing using the Bactec MGIT 960 system. This evaluation was done in the context of time pressures dictated by the need for patients to be evaluated for participation and started on treatment without delay. The observed category agreement between the direct and indirect methods (the reference method or gold standard) was excellent, ranging from 90 to 100% per laboratory. Only 5 discrepant results for 139 pairs (3.6%) were observed, similar to the discordance rate observed previously for direct testing of INH (4.9%) and RIF (3.9%) resistance by Siddiqi et al. (15). The reproducibility of the direct method was excellent, although the numbers are too small to compare and confirm differences.

Compared to standard indirect PZA DST, direct DST was successful in 59% of cases (range across laboratories of 34% to 77%). The reason(s) for the variable performance among all laboratories is inexplicable. Performance was exceptionally poor in one laboratory, with the number of uninterpretable results equally being due to insufficient M. tuberculosis density and contamination. The drug susceptibility testing failures could be attributed to poor techniques for processing of sputum specimens resulting in inadequately digested and decontaminated specimens. Resuspension of the sputum pellet is another critical step to ensure an even distribution of M. tuberculosis bacteria and representative sampling for smear microscopy and culture inoculation. This was the first time that these laboratories performed the direct MGIT drug susceptibility test method, and no on-site training was provided prior to the performance of the study.

The 59% feasibility rate is lower than the rate reported in a recent study where direct susceptibility testing of M. tuberculosis for INH and RIF resistance using the same MGIT system in four laboratories yielded reportable results for 85% of 360 AFB smear-positive sputum specimens (15). As reported previously by Siddiqi et al., the most frequent reason for our uninterpretable direct test results was growth failure. In their study, a 4- to 21-day protocol was used instead of the standard 4- to 13-day protocol for the INH and RIF indirect tests, to allow more time for the growth control tube to reach the required 400 growth units for a valid test. The indirect PZA test protocol is 4 to 21 days long; the extended incubation time allows more time for the M. tuberculosis bacteria to grow if the growth rate in the slightly acidified MGIT PZA medium is lower. The same protocol was used for the direct PZA test since it was not possible to adjust the instrument protocol, i.e., extend it beyond 21 days, using the Bactec MGIT Epicenter system, which was not available in these laboratories. Slow growth of some M. tuberculosis strains in PZA medium may have been a cause for growth failures. It is more likely that the reason for insufficient growth in the control was that the inoculum density was too low. Although the inoculum for the control tube is a 1/10 dilution of the sputum pellet, instead of the 1/100 dilution used in the indirect test, the concentration of viable M. tuberculosis bacteria may have been very low in some sputum specimens despite these specimens being smear positive. Furthermore, it is possible that some strains had a delayed lag time before the beginning of replication and did not reach the threshold of detection before the end of the protocol.

Several approaches for decreasing the number of uninterpretable results can be considered. For the contaminated cultures, the amount of antimicrobial mixture (PANTA), which is added to the control and PZA-containing tubes, could be increased to enhance the suppression of contaminants. To decrease growth failures, a smaller dilution of the sputum sediment could be evaluated as the inoculum for the control, i.e., the use of a 1/5 dilution instead of a 1/10 dilution. Since the number of M. tuberculosis bacteria in the sputum sediment is lower than that in a positive MGIT culture used for indirect testing, the proportions of organisms between the control and drug tubes should still be appropriate with the 1/5 dilution.

The median times for each laboratory to obtain PZA results from the time of specimen collection ranged from 11 to 16 days for the direct test, compared to 18 to 95 days for the indirect test (Table 1). In three laboratories where the direct PZA test was set up within 3 days of specimen collection and results were often available before the end of the 21-day protocol, the turnaround time was 21 days for ≥96% of specimens (Table 2). The longer turnaround time in laboratory 5 was due to the laboratory being busy and prolonging the setup of the direct test. Longer delays were observed for the indirect results when contaminated MGIT cultures had to be decontaminated and recultured and pure M. tuberculosis growth had to be obtained before repeat DST. Logistical problems, such as heavy workload along with insufficient laboratory staff and accessibility to biosafety cabinets, also contributed to the delay in the setup of indirect DST. The time to obtain results after the test was set up ranged from 10 to 16 days for direct tests, compared to 7 to 8 days for indirect tests. A longer time to a result for direct tests is expected, as the inoculum density is smaller, especially for tests that do not reach the growth unit threshold by the end of the 21-day protocol. In a study of INH/RIF direct MGIT testing (15), similar results were obtained: 8 to 14 days for direct test results and 6 to 10 days for indirect test results. However, in the study by Siddiqi et al., the uninterpretable results were not included in the analysis of the time to a positive result (final results). It is likely that uninterpretable results would have a longer time to positive results. Our direct test result times, with and without uninterpretable results, are comparable to those reported for INH and RIF susceptibility testing, suggesting that M. tuberculosis grows at the same rate in MGIT PZA medium as in the MGIT medium used for INH and RIF testing.

Phenotypic methods remain the gold standard for DST in clinical trials, and past and current trials depend on phenotypic testing of anti-TB drugs to ensure that study participants are susceptible to the drugs that they are receiving. Having reliable susceptibility results for the study drugs within the screening period, e.g., 2 to 3 days, would be a significant advancement for clinical trials. Currently, the mechanism or molecular basis of drug resistance is not known for some of the second-line drugs and new TB drugs like bedaquiline, sutezolid, pretomanid (PA-824), and delamanid. Furthermore, not all gene targets associated with resistance are known (e.g., INH, fluoroquinolones, and injectables). Therefore, until current molecular tests are improved or new ones are developed, a rapid phenotypic method like the direct MGIT system would be preferable to indirect MGIT testing. Phenotypic methods may be replaced in the future with molecular tests; however, until we know the relationship between resistance mutations, MICs, and clinical outcomes, there will be a need for phenotypic testing to determine MICs. Rapid MIC determinations are possible with the direct MGIT method (K. Eisenach, unpublished data).

Our study, being conducted in the context of a clinical trial, was limited by the variations in the number and timing of tests in the participating laboratories. However, our results show that once reportable results are obtained, they are reliable and can be obtained in different laboratories. Additional studies with PZA are needed to investigate whether the frequency of uninterpretable results can be decreased by optimizing the method and to gain more experience with MDR-TB/XDR-TB (extensively drug-resistant TB) sputum specimens. If future studies provide reproducible and conclusive data, direct MGIT DST could be the new phenotypic DST standard for clinical trials and clinical management not only for PZA but also for new drugs in clinical development.
